# Fentanyl enhances the excitability of rapidly adapting receptors to cause cough via the enhancement of histamine release in the airways

**DOI:** 10.1186/1745-9974-9-3

**Published:** 2013-02-01

**Authors:** Junzo Kamei, Yuki Nakanishi, Megumi Asato, Hiroko Ikeda

**Affiliations:** 1 Department of Pathophysiology and Therapeutics, School of Pharmacy and Pharmaceutical Sciences, Hoshi University, 4-41, Ebara 2-chome, Shinagawa, Tokyo 142-8501, Japan

## Abstract

**Background:**

Although the mechanism of fentanyl-induced cough is unclear, several lines of evidence suggest that allergic mediators, such as histamine, may play a role in the production of fentanyl-induced coughs. The aim of this study was to explore the effects of fentanyl on cough sensitivity to inhaled citric acid and on histamine release in BALF in mice.

**Methods:**

The cough reflex was induced by the inhalation of citric acid. Male ICR mice were exposed to a nebulized solution of citric acid at a concentration of 0.1 M under conscious and identical conditions using a body plethysmograph. The number of coughs produced per 3-min period of exposure to citric acid was counted. Histamine content in BALF was analyzed by HPLC post-column derivatization and fluorescence detection.

**Results:**

Intravenous administration of fentanyl increased the number of citric acid-induced coughs. The fentanyl-induced enhancement of the number of citric acid-induced coughs was abolished in mice that had been pretreated with moguisteine, a rapidly adapting receptor (RAR) antagonist or fexofenadine, a histamine H1 receptor antagonist. Fentanyl significantly increased the concentration of histamine in BALF.

**Conclusion:**

The results of this study suggest that fentanyl enhances the excitability of RARs to cause cough, and enhancement of histamine release in the airways may some how be related to this change.

## Background

Fentanyl, a synthetic opioid, is a popular choice amongst anesthesiologists because of its quick onset, short duration of action, ease of titrability, intense analgesia, cardiovascular stability and low histamine release [1,2]. Although opioid agonists are known to possess antitussive activity, it is well known that i.v. administration of fentanyl paradoxically induces cough [3-7]. Fentanyl-induced cough may be explosive at times, and may require immediate therapeutic intervention when it undesirably increases intracranial, intraocular or intra-abdominal pressure [5-7]. Although the mechanism of fentanyl-induced cough is unclear, Agarwal et al. reported that pretreatment with salbutamol, beclomethasone or sodium chromoglycate reduced fentanyl-induced coughs [6]. These results strongly suggest that bronchoconstriction and/or allergic mediators, such as histamine and leukotrienes, may play a role in the production of fentanyl-induced coughs.

We previously reported that although the inhalation of 0.1 M citric acid by itself produced only a few coughs in guinea pigs, pre-exposure to histamine concentration-dependently increased the number of citric acid-induced coughs [8]. This histamine-induced increase in the number of citric acid-induced coughs was dose-dependently and significantly reduced when animals were pretreated with fexofenadine, a histamine H1 receptor antagonist. Based on these results, we suggested that histamine enhances the excitability of rapidly adapting receptors in response to tussive stimuli. It is well known that the activation of mast cells and the systemic release of histamine is a common side effect of opioids. While opioids exert antinociceptive effects, less frequent, but perhaps more profound, effects are the bronchoconstriction, edema and hemodynamic instability seen in some patients following intravenous bolus doses of opioids, such as morphine [9-13]. These effects have been attributed to histamine release from mast cells by opioids [9-13]. Although fentanyl has been reported to not cause the release of histamine in plasma [14], no previous study has examined the effects of fentanyl on histamine in bronchoalveolar lavage fluid (BALF).

Therefore, we hypothesized that i.v. fentanyl produces a cough reflex by increasing histamine release in BALF and enhancing cough sensitivity to tussive stimuli. Accordingly, the aim of this study was to explore the effects of fentanyl on cough sensitivity to inhaled citric acid and on histamine release in BALF in mice. The effect of fexofenadine, a histamine H1 receptor antagonist, on the effect of fentanyl on cough sensitivity to inhaled citric acid was also examined.

## Methods

### Animals

Male ICR mice (Tokyo Animal Laboratory Inc., Tokyo, Japan) weighing about 30 g were used. The animals had free access to food and water in an animal room that was maintained at 24 ± 1°C with a 12-h light–dark cycle. These studies were carried out in accordance with the Declaration of Helsinki and/or with the guide for the care and use of laboratory animals as adopted by the committee on the care and use of laboratory animals of Hoshi University, which is accredited by the Ministry of Education, Culture, Sports, Science and Technology, Japan.

### Capsaicin- and citric acid-induced cough reflex

The cough reflex was induced as previously described [8,15]. Briefly, animals were exposed to a nebulized solution of capsaicin at a concentration of 45 μM or citric acid at a concentration of 0.1 M under conscious and identical conditions using a body plethysmograph. The transducer was connected to a respiratory amplifier and polygraph to record a respiratory pattern. The transducer was also connected to respiratory analyzer and to a personal computer, which was used for on-line breath-by-breath measurement. Capsaicin was dissolved to a concentration of 30 mg/ml in a 10% ethanol and 10% Tween 80 saline solution. The stock solution of capsaicin was diluted with saline to a concentration of 15 μM. Citric acid was dissolved to a concentration of 0.1 M in a saline solution. The number of coughs produced per 3-min period of exposure to capsaicin or citric acid was counted.

### Effect of fentanyl on citric acid-induced coughs

The mice were exposed for 3 min to citric acid (0.1 M) 30 min before the i.v. injection of fentanyl (5 μg/kg) to determine the frequency of control coughs. The mice were also exposed for 3 min to citric acid 30 min after the i.v. injection of fentanyl. The number of coughs produced per 3-min period of exposure to citric acid 30 min after the i.v. injection of fentanyl was counted.

### Capsaicin pretreatment

Capsaicin pretreatment was performed to evaluate the possible roles of vagal C-fiber endings and their axonal reflexes in eliciting coughs. A total dose of 100 mg/kg capsaicin was divided into four portions (20, 20, 30, and 30 mg/kg) and injected subcutaneously over 4 days under anaesthesia with ketamine (50 mg/kg, i.m.). Terbutaline (0.1 mg/kg, s.c.) and aminophylline (25 mg/kg, i.p.) were given to counteract the respiratory impairment associated with capsaicin injection. These animals were used for experiments 2 days after the last capsaicin pretreatment.

### Bronchoalveolar lavage fluid (BALF) collection

BALF was collected from the lungs of mice that had been deeply anaesthetized with sodium pentobarbital 30 mg/kg (i.p.). BALF was obtained by injecting 2 ml of ice-cold physiological saline (1 ml, twice) via a tracheal cannula 30 min after i.v. injection of fentanyl (5 μg/kg). The collected BALF (approximately 1 ml) was centrifuged at 1600 × g for 10 min. The supernatant fraction of the collected BALF was stored at −80°C until assayed.

### Histamine assay

Histamine was analyzed by HPLC post-column derivatization and fluorescence detection. The HPLC system consisted of an EP-300 pump (Eicom, Kyoto, Japan), an EHA-500 post-column reagent pump system (Eicom), an ATC-300 column oven (Eicom), a 231XL auto sampler (Gilson, Middleton, USA) and an L-7480 fluorescence detector (Hitachi, Tokyo, Japan). The mobile phase consisted of 0.16 mol/l KH_2_PO_4_ – methanol (97:3, v/v) containing 100 mg/l sodium 1-octanesulfonate (flow rate, 0.3 ml/min). Histamine was separated from other endogenous compounds on a reversed-phase column (Eicompak SC-5ODS; 3.0 mm, i.d. × 150 mm, 5 μm, Eicom) maintained at 42°C. The column elute was mixed with a mixture of 0.05 w/v% *o*-phtalaldehyde (OPA) (flow rate, 0.06 ml/min) and 2.5 mol/l NaOH (flow rate 0.06 ml/min). Histamine was derivatized with OPA to form a fluorescent derivative in a reaction coil (0.25 mm, i.d. × 3 m, PEEK) maintained at 42°C. The reaction mixture was further mixed with 2 mol/l H_3_PO_4_ (flow rate, 0.13 mL/min) in a mixing coil (0.25 mm, i.d. × 2 m, PEEK) to adjust the pH of the final mixture. The fluorescence intensity of the final mixture was monitored at an excitation wavelength of 340 nm and an emission wavelength of 450 nm, and recorded by an EPC-300 data processor (Eicom).

### Drugs

Fentanyl hydrochloride was purchased from Covidien Japan Co. (Tokyo, Japan). Citric acid and capsaicin were purchased from Wako Junyaku Industries Co. (Tokyo, Japan) and Alpus Pharmaceutical Industries Co. (Gifu, Japan), respectively. Fexofenadine hydrochloride was generously supplied by Sanofi-Aventis K.K. (Tokyo, Japan). Histamine hydrochloride was purchased from Sigma-Aldrich Corporation (St. Louis, USA). Methanol and sodium 1-octanesulfonate were purchased from Nacalai Tesque Inc. (Kyoto, Japan). *Ortho*-phtalaldehyde (OPA) was purchased from Wako Pure Chemical Industries, Ltd. (Osaka, Japan). Other chemicals were of the highest purity available and used as received. Distilled water purified with a Milli-Q reagent water system (Millipore, Billerica, USA) was used for all aqueous solutions. Fentanyl and fexofenadine were dissolved in saline and 5% DMSO, respectively. Moguisteine was suspended in 5% carboxylmethyl cellulose (CMC). Capsaicin was dissolved to a concentration of 30 mg/ml in a 10% ethanol and 10% Tween 80 saline solution and was diluted with saline. Moguisteine and fexofenadine was administered s.c. 15 and 30 min before fentanyl injection, respectively.

### Statistics

Data are expressed as means ± S.E.M. The Mann–Whitney *U*-test was used to assess the statistical significance of differences in the number of coughs. Students’ *T* test was used to assess the statistical significance of differences in histamine contents in BALF. A level of probability of 0.05 or less was considered significant.

## Results

### Effects of fentanyl on the number of citric acid-induced coughs

**Figure 1:**
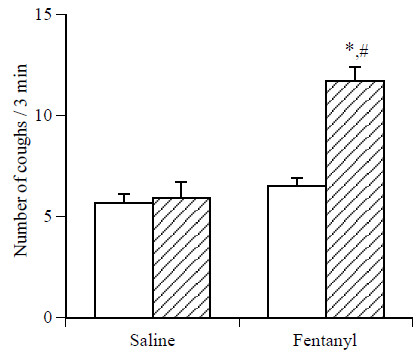
**Effect of fentanyl on the number of citric acid-induced coughs in mice. **The mice were exposed for 3 min to citric acid (0.1 M) 30 min before the i.v. injection of fentanyl (5 μg/kg) to determine the frequency of control coughs (pre-value: open column). The mice were also exposed for 3 min to citric acid 30 min after the i.v. injection of fentanyl. The number of coughs produced per 3-min period of exposure to citric acid 30 min after the i.v. injection of fentanyl (post-value: hatched column) was counted. Each column represents the mean with S.E.M (n = 10). *P < 0.05 vs. respective pre-value. #P < 0.05 vs. respective vehicle (saline)-treated group.

Exposure to citric acid (0.1 M) for 3 min produced about 6 coughs in mice. As shown in Figure 1, while i.v. pretreatment with saline did not significantly affect the number of citric acid-induced coughs, pretreatment with fentanyl at a dose of 5 μg/kg, i.v., significantly increased the number of citric acid-induced coughs in mice.

### Effects of fentanyl on the number of citric acid-induced coughs in C-fiber desensitized (capsaicinized) mice

**Figure 2:**
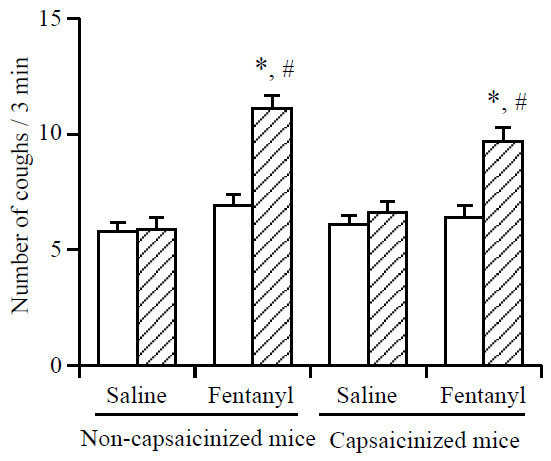
**Effect of fentanyl on the number of citric acid-induced coughs in naive (non-capsaicinized) mice and capsaicinized mice. **The mice were exposed for 3 min to citric acid (0.1 M) 30 min before the i.v. injection of fentanyl (5 μg/kg) to determine the frequency of control coughs (pre-value: open column). The mice were also exposed for 3 min to citric acid 30 min after the i.v. injection of fentanyl. The number of coughs produced per 3-min period of exposure to citric acid 30 min after the i.v. injection of fentanyl (post-value: hatched column) was counted. Each column represents the mean with S.E.M (n = 10). *P < 0.05 vs. respective pre-value. #P < 0.05 vs. respective vehicle (saline)-treated group.

Exposure to capsaicin (45 μM) for 3 min produced 11.6 ± 1.1 coughs (n = 10) in vehicle-treated naive mice (non-capsaicinized mice). On the other hand, the number of coughs was significantly (P < 0.05) reduced (5.6 ± 0.7 coughs, n = 10) when capsaicin-pretreated mice (capsaicinized mice) were exposed to capsaicin (45 μM) for 3 min. As shown in Figure 2, i.v. pretreatment with fentanyl at a dose of 5 μg/kg, significantly increased the number of citric acid-induced coughs in both capsaicinized and non-capsaicinized mice. Pretreatment with capsaicin did not significantly affect the fentanyl-induced enhancement of the number of citric acid-induced coughs.

### Effects of moguisteine and fexofenadine on the fentanyl-induced enhancement of the number of citric acid-induced coughs

**Figure 3:**
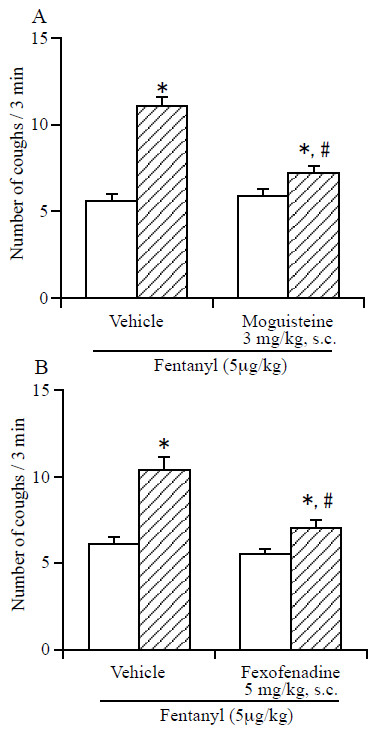
**Effect of moguisteine (A) and fexofenadine (B) on the fentanyl-induced enhancement of the number of citric acid-induced coughs. **The mice were exposed for 3 min to citric acid (0.1 M) 30 min before the i.v. injection of fentanyl (5 μg/kg) to determine the frequency of control coughs (pre-value: open column). The mice were also exposed for 3 min to citric acid 30 min after the i.v. injection of fentanyl. The number of coughs produced per 3-min period of exposure to citric acid 30 min after the i.v. injection of fentanyl (post-value: hatched column) was counted. Fexofenadine (5 mg/kg) and it vehicle (5% DMSO) were administered s.c. 30 min before the i.v. administration of fentanyl. Moguisteine and it vehicle (5% carboxylmethyl cellulose (CMC)) were administered s.c. 30 min before the i.v. administration of fentanyl. Each column represents the mean with S.E.M (n = 10). *P < 0.05 vs. respective pre-value. #P < 0.05 vs. respective vehicle-treated group.

As shown in Figure 3A, the fentanyl-induced enhancement of the number of citric acid-induced coughs was abolished in mice that had been pretreated with moguisteine (3 mg/kg, s.c.), a rapidly adapting receptor antagonist. Pretreatment with fexofenadine (5 mg/kg, s.c.), a histamine H1 receptor antagonist, also abolished the fentanyl-induced enhancement of the number of citric acid-induced coughs (Figure 3B).

### Effect of fentanyl on the level of histamine in BALF

**Figure 4:**
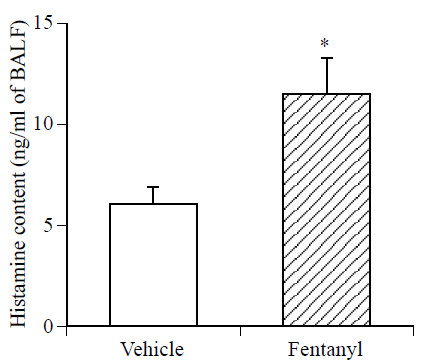
**Effect of fentanyl on the level of histamine in BALF. **BALF was obtained by injecting 2 ml of ice-cold physiological saline (1 ml, twice) via a tracheal cannula 30 min after i.v. injection of fentanyl (5 μg/kg). Each column represents the mean with S.E.M (n = 10). * P < 0.05 vs. vehicle (saline)-treated group.

The concentration of histamine in BALF was significantly increased in fentanyl (5 μg/kg, i.v.)-treated mice compared with that in vehicle-treated mice (Figure 4).

## Discussion

The stimulation of capsaicin-sensitive bronchopulmonary unmyelinated C-fibers is involved in elicitation of the cough reflex [16]. Tachykinins, like substance P, which is contained in afferent C-fiber endings within the airway epithelium and smooth muscle layer, are released by the activation of afferent C-fibers. Bonham et al. reported that substance P stimulates rapidly adapting receptors in guinea pigs [17]. This stimulation of rapidly adapting receptors by substance P is a potential link between the two airway defense systems, both of which elicit bronchoconstriction, mucus secretion and cough. Such a link, by which C-fiber-receptor stimulation leads to the release of substance P and the subsequent stimulation of rapidly adapting receptors with myelinated Aδ fibers, has been previously proposed to explain the overlap of stimuli and reflex effects of both afferent systems [18]; i.e., the activation of C-fibers by capsaicin causes the release of substance P and the subsequent stimulation of rapidly adapting receptors, which may enhance cough reflexes. In the present study, we demonstrated that pretreatment with fentanyl significantly increased the number of citric acid-induced coughs in mice and that this effect was antagonized by pretreatment with moguisteine, a rapidly adapting receptor antagonist. Furthermore, the fentanyl-induced enhancement of the number of citric acid-induced coughs was also observed in capsaicin-pretreated C-fiber desensitized mice. Based on these results, it is likely that fentanyl activates mainly rapidly adapting receptors, but not C-fibers, to enhance citric acid-induced coughs.

In the present study, we also demonstrated that fentanyl-induced enhancement of the number of citric acid-induced coughs was abolished by pretreatment with fexofenadine, a histamine H1 receptor antagonist. However, fexofenadine had no effect on the number of citric acid-induced coughs. Furthermore, in the present study, we also observed that fentanyl significantly increased the histamine levels in BALF. Previously, we demonstrated that pre-inhalation of histamine concentration-dependently and significantly increased the number of citric acid-induced coughs in guinea pigs, and this effect was antagonized by pretreatment with fexofenadine [15]. These results suggest that histamine may enhance cough receptor sensitivity through the activation of histamine H_1_ receptors in the airways. We also observed that the histamine-induced increase in the number of citric acid-induced coughs was concentration-dependently reduced when animals were pretreated with TNP-ATP, P2X-type ionotropic purinergic receptor antagonist [15]. Furthermore, we reported that the ATP-induced increase in the number of citric acid-induced coughs was concentration-dependently reduced when animals were pre-treated with TNP-ATP [19]. We also demonstrated that the ATP-induced increase in the number of citric acid-induced coughs was not abolished in C-fiber-desensitized guinea pigs [19]. On the other hand, inhaled ATP had no significant effect on the number of capsaicin-induced coughs in naive animals [19]. These results suggested that ATP activates C-fiber-independent pathways, i.e., it directly activates rapidly adapting receptors, to enhance citric acid-induced coughs [19]. Tamesue et al. reported that histamine induced the release of ATP from segments of vas deferens, and this effect was blocked by pyridamine and triprolidine, histamine H_1_ receptor antagonists, but not by ranitidine, an H2 receptor antagonist [20]. Furthermore, histamine also increased the release of ATP from superfused cultured smooth muscle cells [20]. The authors suggested that ATP might be released as an autacoid from smooth muscles in the presence of histamine [20]. Taken together, these results suggest that although histamine, by itself, does not directly modulate the sensitivity of the cough reflex, it is likely to enhance the excitability of rapidly adapting receptors and/or cough receptors in response to tussive stimuli via the modulation of ATP release in the airways. Based on our previous and present results, it is possible that fentanyl enhances the release of histamine in bronchoalveolar tissue and this histamine first enhances the excitability of rapidly adapting receptors through the activation of histamine H1 receptors and then causes a cough reflex.

Narcotic analgesics can induce the release of histamine both in vitro and in vivo in animals as well as in human. However, plasma histamine levels after i.v. injection of fentanyl are much less than those after the injection of morphine [1,2,14]. Furthermore, incubation of human skin mast cells with fentanyl did not induce the release of histamine [21]. However, in the present study, i.v. injection of fentanyl markedly increased the histamine levels in BALF. Although further studies are needed to determine the mechanisms that underlie these findings, it is possible that mast cell heterogeneity may play a role in the different effects of fentanyl on histamine release.

In conclusion, our present results suggest that fentanyl enhances the excitability of rapidly adapting receptors to cause cough. Furthermore, enhancement of histamine release in the airways might some how be related to fentanyl-induced enhancement of cough sensitivity. However, further studies are necessary before this possibility can be established with greater certainty.

## Competing interests

All authors declare that they have no competing interests.

## Authors’ contributions

YN, HI and MA conducted many of the experiments, analyzed the data and created the graphic summaries of the results. JK is general conductor of this study and wrote the manuscript. All authors read and approved the final manuscript.
